# Biology of pancreatic stellate cells—more than just pancreatic cancer

**DOI:** 10.1007/s00424-017-1968-0

**Published:** 2017-04-05

**Authors:** Pawel E. Ferdek, Monika A. Jakubowska

**Affiliations:** Medical Research Council Group, Cardiff School of Biosciences, Cardiff University, Cardiff, Wales CF10 3AX UK

**Keywords:** Pancreatic stellate cells, Fibrosis, Pancreatitis, Pancreatic cancer, Calcium, Myofibroblasts

## Abstract

Pancreatic stellate cells, normally quiescent, are capable of remarkable transition into their activated myofibroblast-like phenotype. It is now commonly accepted that these cells play a pivotal role in the desmoplastic reaction present in severe pancreatic disorders. In recent years, enormous scientific effort has been devoted to understanding their roles in pancreatic cancer, which continues to remain one of the most deadly diseases. Therefore, it is not surprising that considerably less attention has been given to studying physiological functions of pancreatic stellate cells. Here, we review recent advances not only in the field of pancreatic stellate cell pathophysiology but also emphasise their roles in physiological processes.

## Introduction

The diffuse stellate cell system is composed of star shaped cells woven into various mammalian organs including, but not limited to, the liver, pancreas or kidney [26, 32, 136]. Despite some tissue-specific differences, so-called quiescent stellate cells are uniformly characterised by their ability to store retinoids—vitamin A and its analogues—mainly in a form of lipid droplets scattered in the cytosol [2, 34, 136]. These lipid-packed cells normally possess only a limited capacity to proliferate and migrate within the parenchymal tissue and show no detectable expression of α-smooth muscle actin (α-SMA) [2, 32]. Importantly, loss of retinoid droplets, along with increased expression of α-SMA, is a concomitant of stellate cell transition to an activated myofibroblast-like phenotype [26] in response to inflammatory or carcinogenic processes [5, 8, 122]. As a result, activated stellate cells not only become capable of intensive proliferation and migration, but also get heavily involved in the extracellular matrix (ECM) protein turnover, contributing towards tissue remodelling [5]. However, continued tissue injury may interfere with the normal healing processes, leading to an extended presence of activated stellate cells and resulting in excessive tissue scarring [113]. Interestingly, this may impact not only on physiological functions of the affected tissue but also on its biomechanical properties [101]. For example, stellate cell-mediated fibrosis of the vocal folds could impair the normal tissue flexibility required for emission of voice [35], a problem not uncommon for singers or broadcast personnel.

In the pancreas, pancreatic stellate cells (PSCs) build up only about 4–7% of the organ [2] and, in contrast to the more abundant acinar cells or islets, neither secrete digestive enzymes nor hormones. However, in chronic pancreatitis and pancreatic ductal adenocarcinoma (PDAC), it is the activated PSCs that deposit collagen fibres and contribute to the development of pancreatic fibrosis [5, 26]. Activated PSCs have recently been the focus of multiple studies and continue to attract a lot of interest, especially in relation to pancreatic cancer, often perceived as a death sentence. PSCs have not only been shown to form a dense fibrotic stroma and interact with cancer cells, but may also be capable of travelling within the body to colonise distant metastases [122, 131]. Despite this substantial progress made in the past two decades, to date surprisingly little is known about the physiological roles of quiescent PSCs in the healthy tissue. Here, we highlight the advances in the PSC field, predominantly in respect of the function of these cells in the normal tissue, their roles in acute and chronic pancreatitis as well as in pancreatic cancer. Also, we would like to draw particular attention to the involvement of ion channels in PSC biology.

## Discovery

Although the discovery of hepatic stellate cells (HSCs) is commonly attributed to Carl von Kupffer [32], who also introduced the term “stellate cells” (1876), more than one research group contributed to the identification of PSCs. The first documented observation most likely describing what we know today as PSCs was published by Watari et al. in 1982 [126]. In the pancreata isolated from mice fed with retinoids, the authors noticed vitamin A-loaded cells either scattered randomly in the tissue or located in the vicinity of the blood capillaries [126]. Even though not explicitly referred to as PSCs, periacinar fibroblast-like cells were first isolated and cultured in 1997 by Saotome et al. [99]. However, most of the credit for identification of PSCs has been given to two independent research papers accepted for publication a year later [2, 6]. Both those studies, by Apte et al. and Bachem et al. [2, 6], applied density gradient centrifugation to isolate quiescent rat PSCs, a procedure previously used for purification of HSCs [33, 34, 66]. Bachem et al. also introduced the outgrowth method that yielded activated PSCs, neatly grown out of small tissue blocks of either rat or human origin [6]. PSC research has been further aided by the development of an immortalised rat cell line in 2004 [113]. These studies triggered a sudden outburst of interest in the previously overlooked cells that continues until today.

## Pancreatic versus hepatic stellate cells

PSCs are often compared to HSCs due to their similar morphological and functional features. In principle, both cell types are capable of expressing the same protein markers such as desmin and glial fibrillary acidic protein (GFAP); however, the exact expression levels vary markedly between different species [36, 104, 132, 136] or even in different cells of the same individual [45]. The gene expression profiles of PSCs and HSCs show a high degree of similarity, but differ from fibroblasts [14]. In contrast to stellate cells in their activated phenotype, fibroblasts are generally negative for desmin and α-SMA and also show a less pronounced synthesis of ECM proteins [7].

Despite clear similarities, some organ-specific differences in expression patterns exist between PSCs and HSCs. To name a few, PSCs are characterised by higher levels of α7-integrin, hypoxia inducible factor 1α subunit (HIF1α), and cytoskeletal components [14]. Therefore, findings related to one cell type cannot be ad hoc transferred to another.

Since PSCs express both mesenchymal and ectodermal markers, their origin has been the subject of debate. A similar discussion has been finally settled for HSCs owing to a study that pointed towards their mesenchymal origin [16]. This is also likely to be true for PSCs, but firm experimental evidence is still lacking. Nevertheless, at least a subpopulation of PSCs in the normal and inflamed pancreas has been shown to be derived from the bone marrow progenitors [73, 102, 114].

## Quiescent pancreatic stellate cells

**Fig. 1:**
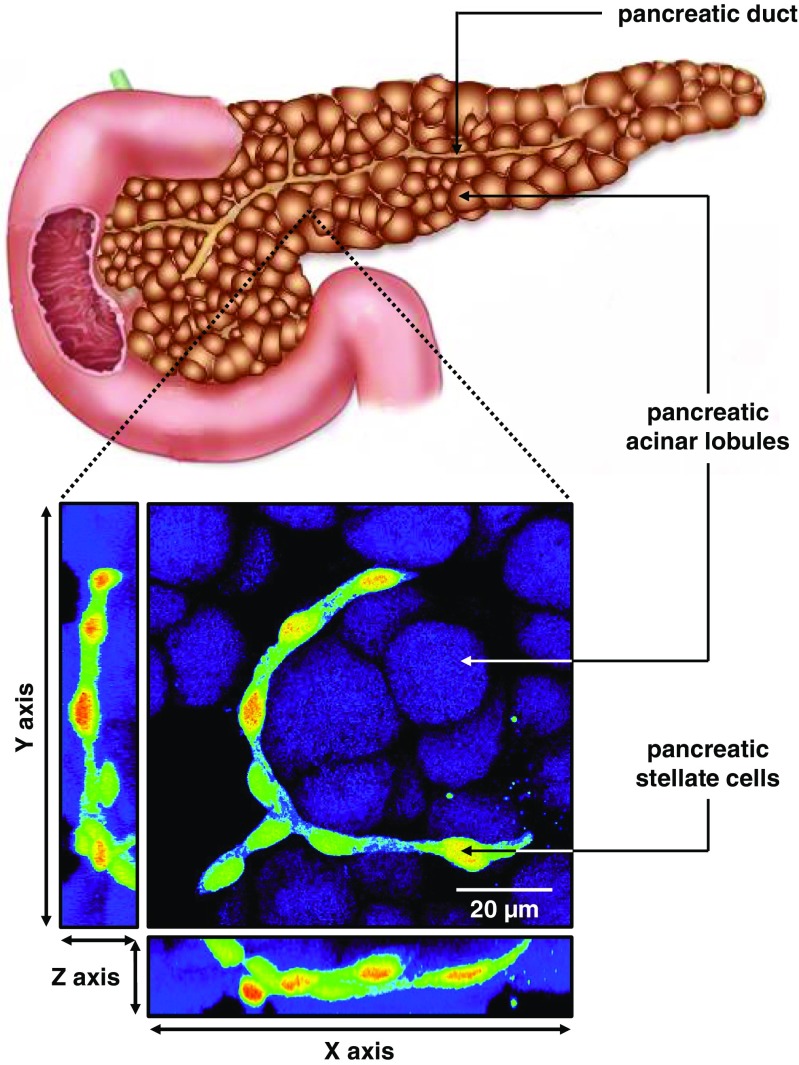
Schematic illustration of the pancreas. The exocrine part of the organ predominantly consists of acinar lobules. Pancreatic stellate cells (shown in *bright green-yellow-red pseudocolours*, lower panel) form a three-dimensional network in between those lobules (*dark purple*)

In their quiescent phenotype, PSCs appear stagnant and almost redundant and currently very little is known about their physiological functions. These cells normally form a three-dimensional network that runs in between pancreatic lobules (Fig. 1) adjacent to the ducts and blood capillaries [2]. Interestingly, it remains unexplored whether normal functioning of PSCs depends on maintaining this characteristic network-like structure. The presence of stellate cells has also been reported in islets of Langerhans, predominantly responsible for the release of insulin and glucagon [134]. It was suggested that these particular cells may be a subpopulation of conventional PSCs [134] that play a role in islet fibrosis related to severe cases of diabetes [51].

Quiescent PSCs are most likely responsible for the turnover of ECM components since they secrete metalloproteinases (MMP) including MMP-2, MMP-9, and MMP-13, as well as their inhibitors [95]. That, however, is rather unlikely to exhaust the full array of physiological functions of quiescent PSCs. Other roles for these cells have been postulated, such as the cholecystokinin (CCK)-elicited release of acetylcholine (ACh), which in turn stimulates acinar secretion [96]. Cultured human PSCs were shown to express ACh synthesising systems and CCK receptors [96]. However, experiments on isolated pancreatic lobules provided no evidence for the presence of CCK receptors in mouse quiescent PSCs; also no Ca^2+^ responses were detected in those cells upon CCK stimulation [40]. Furthermore, since expression of toll-like receptors (TLR) have been found in isolated rat PSCs, one might speculate that stellate cells could play a role in innate immunity by phagocytosis of exo- and endogenous antigens [81]. This notion is further supported by a different study, which demonstrated phagocytic activity in PSCs as well as the presence of the scavenger receptor CD36 [110], also known to be expressed by monocytes/macrophages [89]. Interestingly, in HSCs, this receptor is fully functional and capable of binding oxidised low density lipoprotein, which is associated with acquisition of the activated phenotype [63, 106]. This may suggest some similarities of stellate cells to phagocytic immune cells.

**Fig. 2:**
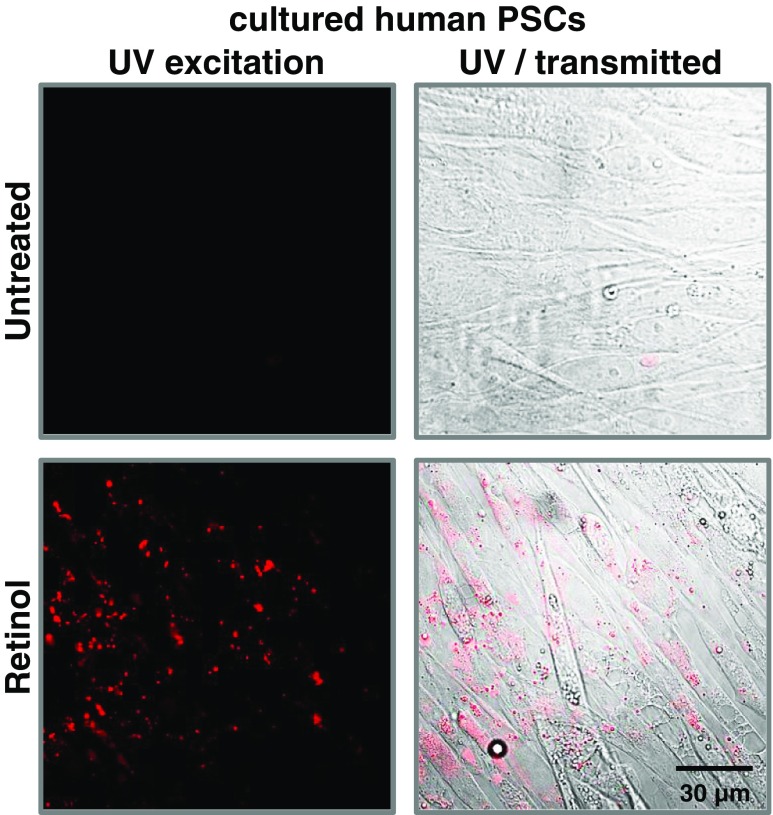
Pancreatic stellate cells have the capacity to store retinoids. Cultured human PSCs quickly become activated and lose most of their stored retinol (upper panel). In the presence of 100 μM retinol (24 h treatment), lipid vesicles appear in the cytosol of PSCs (lower panel, *red pattern*), which is revealed by excitation with UV light

Quiescent stellate cells contain retinoids (Fig. 2), predominantly as retinyl palmitate cytosolic droplets [11, 48], whose formation is likely to be dependent on intracellular albumin [65]. In the adult body, up to 80% of dietary retinoids is stored in the liver [12], in which HSCs remain a fraction 60-times enriched in vitamin A analogues as compared to the liver parenchymal cells [12]. The levels of retinoids contained in PSCs are substantially lower and more variable than in HSCs [55]. The exact role of retinoids in PSCs has not been investigated in great detail. It is well known, however, that retinoid family members are vital for the maintenance of tissue homeostasis by controlling cell growth, differentiation as well as apoptotic cell death [9, 98, 118]; whereas by regulating embryonic signalling pathways [20, 97] they govern “stemness” of cancer cells [9, 133]. During early days of development in utero*,* the gradient of retinoid distribution may serve either as an instructive or permissive signal for embryogenesis [24]. Retinoic acid (RA) is required for normal development of the embryonic pancreas [24, 97], as shown in the frog [18], zebra fish [53], and mouse models [74]. Further, the influence of retinoids on the organogenesis of the pancreas is related to their stimulatory effect on differentiation of endocrine and duct cells [53, 118], and apoptosis of acinar cells [118]. In adult pancreas, RA isomer 9-*cis*-retinoic acid (9cRA) has been shown to act as a pancreas-specific autacoid [62]. As it has been demonstrated, 9cRA is generated in situ in the pancreas, where it briefly attenuates glucose-stimulated insulin secretion [62].

Retinoids may facilitate maintenance of the quiescent state of PSCs, especially given that retinol and its metabolites have been shown to inhibit expression of α-SMA and decrease activation of relevant signalling pathways [82]. *All-trans*retinoic acid (ATRA) has been proposed to trigger restoration of mechanical quiescence of PSCs [19, 100], suppress their capacity to remodel the extracellular matrix [100] and thus inhibit cancer cell invasion [19].

## Activated pancreatic stellate cells

The pathophysiological roles of PSCs become apparent in healing injuries caused by inflammation. Despite having certain stem cell characteristics [26], PSCs probably cannot directly replace or regenerate damaged cells; instead they substitute lost cellular components with fibrotic tissue. This “quick fix” is initially crucial for restoring organ integrity. However, an extended presence of activated PSCs may transform into a pathological process leading to the deposition of excessive amounts of ECM proteins and thus permanent scarring of the pancreas accompanied by loss of cellular components.

Damage to the pancreatic tissue triggers activation of PSCs, in response to inflammatory mediators [1, 83], alcohol metabolites [4] or growth factors such as the platelet-derived growth factor (PDGF) [3, 105] or transforming growth factors TGF-α and TGF-β [3, 116, 121]. These activating factors are present in the inflamed pancreas and are secreted by infiltrating cells (e.g. macrophages), platelets, endothelial cells or pancreatic acinar cells [75]. Also, transformed cells in pancreatic cancer are a source of agents triggering activation of PSCs [26]. Importantly, PSCs themselves may be able to secrete certain growth factors (e.g. PDGF) or cytokines and thus facilitate their activation in auto- or paracrine manner [3, 70, 105, 109].

**Fig. 3:**
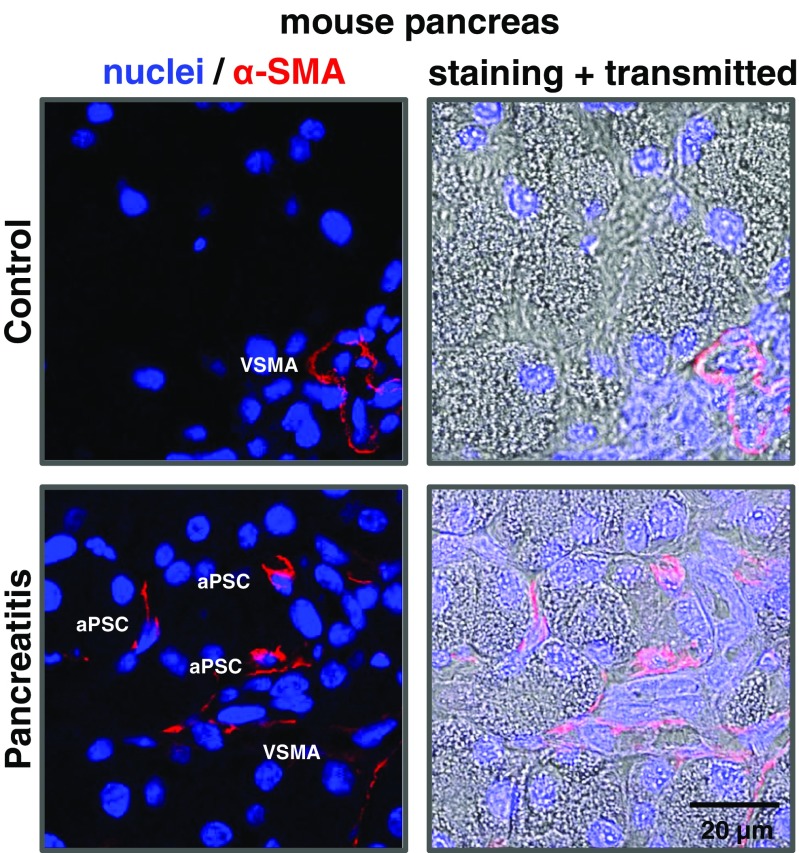
Expression of α-smooth muscle actin (α-SMA), and thus the number of activated PSCs (aPSCs), increases as a result of tissue inflammation. In the healthy mouse pancreas (upper panel) α-SMA-positive staining (*red*) is only present in the vascular smooth muscle cells in the blood vessels and is labelled as vascular smooth muscle actin (VSMA). Induction of pancreatitis (by ethanol and fatty acids) leads to a sudden appearance of α-SMA-positive cells—aPSCs—scattered within the parenchymal tissue

The process of PSC phenotype transition is associated with clear morphological and functional changes. Its most widely accepted features are loss of retinoid droplets from the cytosol and increased expression of α-SMA (Fig. 3) [2, 6]. Activated PSCs assume a spindle-like shape in vitro, actively proliferate and migrate as well as show an increase in production of collagen type I and III, laminin and fibronectin [6]. Furthermore, they also secrete neutrophil chemotactic factor IL-8 and macrophage chemoattractant protein-1 (MCP-1) [1, 117]; as well as express intracellular adhesion molecule-1 (ICAM-1) [77]. This suggests that activated PSCs may be involved in exacerbating inflammation in the pancreas by recruitment of inflammatory cells. The presence of α-SMA along with endothelin-1 gives PSCs elasticity and the potential for contractions [79]. Given the periductal and perivascular localisation of these cells, it has been speculated that the contractility traits of activated PSCs may contribute to the regulation of vascular and ductal tones [75]. Also, PSCs in their myofibroblast-like phenotype have been implicated in the remodelling and further stiffening of pathological fibrosis in response to external mechanical stimuli [19]. Processes of PSC activation may thus affect the biomechanical tissue homeostasis.

A detailed clarification of the mechanisms controlling phenotype transition of PSCs probably represents one of the most important challenges in the stellate cell field in the coming years. Among the identified candidates implicated in this process are the mitogen-activated protein kinase (MAPK) family members [60, 78], NF-κB [77, 109] and downregulation of peroxisome proliferator-activator receptor γ (PPAR-γ) [61, 76]. Signalling pathways associated with the phenotype transition have been reviewed in more detail in previous publications [59, 75].

## Ion channel biology of pancreatic stellate cells

The importance of Ca^2+^ signalling in the exocrine pancreas is well illustrated by the fact that secretion of digestive enzymes by acinar cells is controlled by intracellular Ca^2+^ oscillations [91, 93]. What is more, dysregulated Ca^2+^ signals underlie the necrotising diseases of the pancreas: acute and chronic pancreatitis [38, 92]. Although Ca^2+^ signalling events have been extensively investigated in pancreatic acinar cells, ion fluxes in PSCs and their consequences have been a subject of only a very limited number of studies. However, as discussed below, recent evidence revealed that the physiology of PSCs is also regulated by intracellular Ca^2+^ signalling and further insights into these processes may shed new light on the roles of quiescent PSCs and the mechanisms of their phenotype transition.

The first attempt to characterise the differences in Ca^2+^ signals between quiescent and activated PSCs has been made by Won et al. [128]. Their work elegantly demonstrated that while activated PSCs responded to agonists of protease-activated receptor 1 or 2 (thrombin and trypsin, respectively) with transient elevations of intracellular Ca^2+^, these responses were completely absent in quiescent PSCs [128]. Importantly, the authors also showed that angiotensin and bradykinin were potent inducers of Ca^2+^ signals in both quiescent and activated PSCs [128]. A later study by Gryshchenko et al. revealed that bradykinin receptor 2 was responsible for the bradykinin-induced intracellular Ca^2+^ elevation in these cells [40]. Expression of this receptor has been shown both in paraffin-embedded mouse pancreatic tissue slices [30, 58] and in cultured human PSCs [30]. Bradykinin responses could be used to distinguish PSCs from acinar cells (unresponsive to bradykinin) and thus may be a useful physiological marker of the stellate phenotype in the pancreatic tissue [30, 58].

Several types of purinergic receptors P2X and P2Y have been found in PSCs [15, 49, 68] and Ca^2+^ responses to ATP, UTP and UDP have been recorded in these cells [49]. Won et al. demonstrated that activated PSCs were more sensitive to ATP than the quiescent cells [128]. This is interesting, especially in light of the findings of Haanes et al., who showed that high ATP concentrations induced cell death in normal PSCs but not in cells lacking the functional purinergic receptor P2X7 [42]. The authors concluded that the latter receptor may act as a death receptor when exposed to high doses of ATP [42]. It is therefore likely that ATP could contribute to removal of activated PSCs. Also, the P2X7 receptor has been suggested to be involved in regulation of PSC proliferation, since mice lacking this receptor had substantially fewer PSCs than normal mice; and those cells proliferated more slowly in culture than normal cells [42]. Of note is also the observation that low ATP levels had a stimulatory effect on proliferation of PSCs [42].

Zhang et al. reported increased expression of the transient receptor potential vanilloid 4 (TRPV4) channel in PSCs isolated from rats fed with high-fat and alcohol diet for 6 weeks [135]. Despite the mild phenotype of chronic pancreatitis (as shown by histological techniques), increased and sustained intracellular Ca^2+^ mobilisation was observed. The authors concluded that TRPV4 is a functional ion channel in PSCs, which mediates responses to metabolites of alcohol and fatty acids [135].

A very recent study found a potentially important link between Ca^2+^ fluxes and pancreatic cancer desmoplasia, which contributes to increased physical pressure in the neoplastic tissue. This high pressure led to activation of mouse PSCs, a process mediated by Ca^2+^ influx through the transient receptor potential canonical 1 (TRPC1) channels; and thus the authors suggested a link between TRPC1 and pressure sensing in PSCs [29].

The importance of ion channels has also been illustrated by another study that not only provided the evidence for functional expression of the Ca^2+^ sensitive K^+^ channel of intermediate conductance, K_Ca_3.1, and the TRPC3 channel in human PSCs, but also demonstrated their role in PSC migration [115]. The distribution patterns of these two channel proteins in the plasma membrane of human PSCs revealed a very high degree of colocalisation [115]. The authors postulated cooperation between the two channels, which was based on the fact that Ca^2+^ responses in PSCs, induced by PDGF, were reduced by pharmacological inhibition of K_Ca_3.1 channels and completely abolished by the knockdown of TRPC3 [115]. Both inhibition of K_Ca_3.1 and loss of TRPC3 channels substantially decreased PSC migration [115]. Given that the inhibition of Ca^2+^ channels has already been demonstrated to be beneficial in acute pancreatitis [37, 127], analogous strategies could be employed in novel therapeutic approaches against chronic pancreatitis and pancreatic cancer, targeting ion channels that drive PSC migration and proliferation.

In the study of Kemeny et al. [64], myofibroblasts have been isolated from different tissues of the human gastrointestinal tract and showed remarkable similarities in the expression patters of α-SMA, desmin, vimentin and cytokeratin to PSCs. Interestingly, human gastric myofibroblasts were demonstrated to express all three isoforms of Na^+^/Ca^2+^ exchanger (NCX), which was attributed to the regulation of Ca^2+^ homeostasis in these cells as well as migration and proliferation [64].

Finally, experiments on mouse pancreatic lobules demonstrated that Ca^2+^ responses, induced in PSCs by both physiological and pathophysiological stimuli, do not propagate to the adjacent acinar cells [30, 40, 58]. Therefore, gap junctions, well known for allowing communication and signal propagation between acinar cells [56, 57], are unlikely to exist between acinar cells and PSCs [41].

## Pancreatic cancer

Pancreatic cancer affects almost 340,000 people worldwide annually and is highly resistant to chemotherapy, which results in a devastating prognosis for the patients: the median life expectancy of about 6 months post diagnosis [120] and the 5-year survival rate below 5% [124]. Infamously known as “partners in crime” [122], PSCs have recently been in the spotlight owing to their involvement in pancreatic cancer aetiology. Importantly, they have been postulated not only to contribute to the development of solid pancreatic tumours [54, 112], of which PDAC is the most common [50], but also to facilitate spreading of the disease by supporting formation of the secondary tumours (metastases) in the distant locations [43, 107, 122, 131]. In addition, a recent study has proposed that PSCs may be mediators of pain in pancreatic cancer [44]. Of note is that also somewhat conflicting evidence exists in the literature, whereby depletion of α-SMA-positive myofibroblasts and thus reduced desmoplastic reaction in mouse models of PDAC, resulted in adverse outcomes, including impaired immune response and decreased survival [86].

Associated with pancreatic cancer, fibrotic stroma comprises PSCs and the products of their secretion, and may account for even up to 80% of the tumour mass [27]. A complicated network of interactions between cancer cells and PSCs has been shown to perpetuate the desmoplastic reaction [5, 8, 107], in which the growth of the fibrotic tissue is induced by a primary distortion in the organ [5]. This leads to the formation of the collagen-rich fibrotic microenvironment, which tightly surrounds the malignant cells [8, 107] and thus may restrict blood flow, availability of oxygen, as well as limit inflammatory infiltration [28] and the delivery of chemotherapeutic agents [21, 108]. Notably, the cross-talks between cancer cells and PSCs may result in further remodelling of the stromal microenvironment via activity of MMP-2. MMP-2-mediated degradation of the stromal proteins promotes invasiveness and tumourigenicity of cancer cells, as was shown by assessment of cancer cell migration or formation of xenograft tumours in an immunodeficient mouse host [107]. The extracellular matrix may also resemble a reservoir of sequestrated mediators of inflammation released upon tissue stress or injury [103] as happens in diseases of the pancreas: pancreatitis and pancreatic cancer [52, 103]. The MMP-family enzymes have been implicated in inflammatory mechanisms, wherein they serve as damage-associated molecular patterns (DAMPs) [103].

Poor oxygenation (hypoxia) and limited nutrient availability are the hallmarks of solid tumours [39], including PDAC. The hypoxic stromal environment provides a selective pressure for the expansion of mutant cells of abnormal signalling and proliferative capacity. This may translate into tumour resistance to therapeutic approaches [39], including radiation [25]. PSCs have been shown to radioprotect the cancer cells through a β1-integrin pathway; whereas tumours without PSCs responded to radiotherapy with a delayed growth and decreased volume compared to the tumours consisting of both cancer cells and PSCs [72].

Hypoxic conditions have also been suggested to stimulate expression of angiogenesis-regulating molecules in PSCs [80, 131]. This may play a role in spreading of the cancer cells via the blood stream or lymphatic circulation, and further progression of the cancer. Indeed, PSCs have been found to induce formation of metastases [54]. Even more importantly, a sex-mismatch study elegantly proved the ability of PSCs to accompany pancreatic cancer cells to the metastatic sites [131]. In that work, orthotopic xenograft tumours in the pancreata of female mouse hosts were formed out of both human male PSCs and human female pancreatic cancer cells. This experimental setup allowed the authors to use the Y chromosome as a marker of PSCs, identified by fluorescent in situ hybridisation [131]. This confirmed the presence of exogenously introduced PSCs not only in the primary tumours in the pancreas but also in the metastases in the liver, mesentery and thoracic diaphragm [131]. Noteworthy, PSCs alone did not form tumours during a 6-month period post injection [72].

PSCs not only provide the ideal environment for the development of pancreatic cancer, protecting it against the anti-cancer therapies and facilitating its spreading, but also may “feed” the tumour. A recent study has shown that PSCs are critical for PDAC metabolism through the secretion of non-essential amino acids [112]. These amino acids, especially alanine, have been postulated to be an alternative source of carbon for the tricarboxylic acid cycle in the PDAC cells, that experience shortage of glucose and glutamine-derived carbon due to the surrounding stroma [112]. Interestingly, alanine secretion by PSCs is dependent on their autophagic death stimulated by the cancer cells [112]. Targeting such cross-talks between PSCs and cancer cells is an emerging novel therapeutic strategy against PDAC.

## Pancreatitis

Chronic pancreatitis becomes increasingly common in the developed countries and it is generally agreed that alcohol plays a significant role in its pathogenesis [71]. Despite intensified research, still there is no effective treatment other than supportive care. Generation of reactive oxygen species and fatty acid ethyl esters, as a result of ethanol metabolism [87], induce injury of the tissue predominantly by triggering abnormal Ca^2+^ signals in acinar cells along with a decrease in ATP levels, followed by acinar necrosis [92, 94]. Chronic inflammation, oxidative stress and ethanol metabolites interfere with the normal healing processes [129] leading to prolonged activation of PSCs that replace acinar cells and pancreatic islets by non-cellular fibrotic tissue. This impairs both exocrine and endocrine functions of the pancreas, often resulting in malnutrition and diabetes [13]. Although not explicitly described as activated PSCs, substantial quantities of α-SMA-positive myofibroblasts were found in alcoholic pancreatitis in human patients [22] and activated PSCs are a typical feature of animal models of chronic pancreatitis [69]. Repetitive pancreatic injury, induced by cerulein (a compound similar in action to CCK), causes deposition of collagen, and PSCs were found to be its major source [17, 85].

Accumulating data indicates that pancreatic fibrosis can be reversed, at least in the early stages of chronic pancreatitis [123]. It was also demonstrated that administration of RA can supress the deposition of collagen fibres [130]. However, it remains unknown if this regression of pancreatic fibrosis is dependent on transition of PSCs back to the quiescent phenotype. In fact, it is not entirely clear whether PSCs are able to revert to quiescence in vivo. Instead, they could be regenerated from a population of PSCs that have not undergone activation during injury or from a pool of progenitor cells [125]. Therefore, the phenomenon of a phenotype transition in PSCs may hold the key to our understanding of the mechanisms that drive pancreatic fibrosis and could be a viable target in anti-fibrotic therapies.

**Fig. 4:**
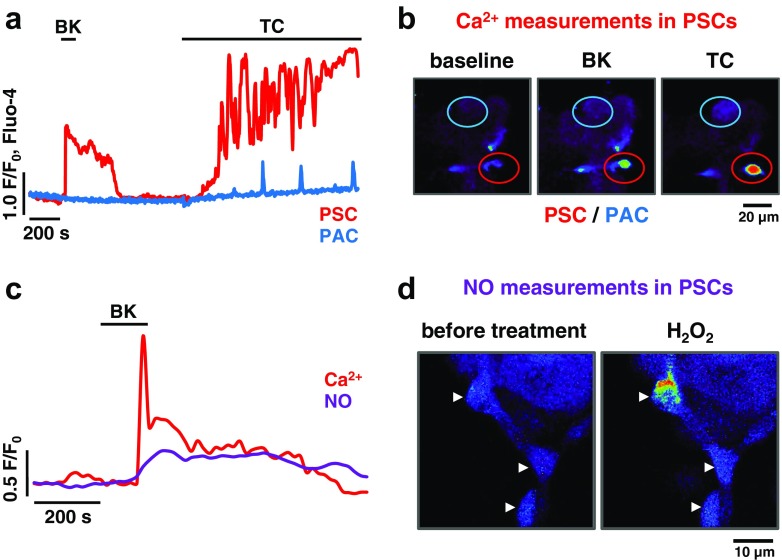
Mouse pancreatic stellate cells, in their native environment of pancreatic lobules, respond to pathophysiological stimuli with intracellular Ca^2+^ signals as well as generation of NO. **a** Sample traces recorded in mouse pancreatic lobules loaded with a Ca^2+^-sensitive dye Fluo-4 AM. Pancreatic stellate cell (PSC, *red trace*) responds to 10 nM bradykinin (BK) but pancreatic acinar cell (PAC, *blue trace*) does not, which confirms the stellate phenotype. The PSC subsequently responds to 5 mM taurocholate (TC) with a large elevation of intracellular Ca^2+^, whereas the neighbouring PAC generates only modest Ca^2+^ oscillations. For more information, the reader is referred to a study by Ferdek et al. [30]. **b** Individual images from the recording shown in (**a**). The *red circular regions* mark the PSC that responded to bradykinin and then to taurocholate with increases in intracellular Ca^2+^ concentration. The *blue circular regions* indicate the PAC that did not respond to bradykinin and produced only transient Ca^2+^ elevations in response to treatment with taurocholate. **c** Sample traces recorded in a PSC embedded in a mouse pancreatic lobule loaded with both Fura-2 AM (Ca^2+^-sensitive dye) and DAF-2 (NO-sensitive dye). The cell responds to 20 nM BK with an elevation of intracellular Ca^2+^ concentration (*red trace*) and a simultaneous increase in intracellular NO (*purple trace*). For more information, the reader is referred to a study by Jakubowska et al. [58]. **d** Sample images show a mouse pancreatic lobule, loaded with DAF-2, before and after treatment with 500 μM hydrogen peroxide (H_2_O_2_). PSCs are indicated with *white arrowheads*. Treatment with H_2_O_2_ increases intracellular NO in these cells (shown as a shift in the pseudocolour spectrum)

Migrating gallstones can cause bile reflux into the pancreas, which induces severe inflammation of the organ. While the bile is the most common cause of acute pancreatitis, its capacity to induce the chronic, and thus fibrotic, form of the disease is marginal [10, 88]. In a recent study, it was reported that mouse PSCs, located in their native environment, were remarkably sensitive to the most common bile components [30]. Bile acids, sodium cholate and taurocholate, caused large and sustained Ca^2+^ signals in the cytosol of PSCs, quickly followed by necrotic death, whereas the effects of those bile acids on neighbouring acinar cells were much less prominent (Fig. 4a, b) [30]. Interestingly, PSCs appear to utilise specific mechanisms of bile acid uptake resembling those present in the liver [30]. These results are particularly surprising as, according to the prevailing dogma, the adverse effects of the bile were predominantly attributed to premature activation of digestive enzymes in acinar cells, a process triggered by excessive intracellular Ca^2+^ signals [38, 94]; and, to a lesser extent, impaired ductal secretion [47, 119]. Therefore, the recent report sheds new light on the pathogenesis of biliary pancreatitis, whereby bile acids are likely to deprive the pancreas of its repair mechanisms driving up the severity of the disease in the initial stages. At the same time, by killing PSCs, in a Ca^2+^-dependent manner, bile acids may not promote the development of pancreatic fibrosis in the long term. This discovery suggests that certain bile acids could even be used as therapeutic agents against fibrosis [46].

Of note is that the effects of the bile acids were further exacerbated by a pro-inflammatory mediator bradykinin [30]. Injury to acinar cells causes release of enzymes stored in zymogen granules, including trypsin and kallikreins, which in turn, act on kininogens to generate kinin peptides (such as bradykinin) and further escalate the on-going inflammatory processes [41]. Indeed, increased concentrations of bradykinin elicit Ca^2+^ responses in PSCs, that may lead to their activation and proliferation [41].

Another study has shown that the bile acid-induced pathophysiological Ca^2+^ signals in PSCs, but not in acinar cells, are accompanied by nitric oxide (NO) generation [58]. In addition, bradykinin (Fig. 4c) and hydrogen peroxide (Fig. 4d) have been demonstrated not only to cause intracellular Ca^2+^ elevation but also a simultaneous increase in NO production in PSCs [58]. This indicates a link between the two signalling pathways. Expression of inducible NO synthase (NOS2) is present in PSCs, as shown by colocalisation with bradykinin receptor type 2 [58]. This is similar to a previous work that indicated NOS2-dependent production of NO in α-SMA- and vimentin-positive pancreatic myofibroblasts that well could have been PSCs [84]. However, the actual role of NO in pancreatic diseases remains ambiguous. On the one hand, reactive oxygen/nitrogen species, such as NO, are present in the inflamed tissue and may chemically modify cellular components [111]. Importantly, inhibition of NO generation has been demonstrated to protect both PSCs and adjacent acinar cells against necrosis [58]. On the other hand, vascular tone and pancreatic secretion were suggested to be regulated by NO [67, 90], whose production was previously attributed only to endothelial cells in the pancreas [67]. Given the recent data demonstrating that PSCs can also produce NO, PSCs may contribute to the local control of circulation and secretion in the organ. Furthermore, in pancreatitis, the overproduction of NO by PSCs, in response to bile acids or bradykinin, may play a role in the increased vasodilation of ducts and blood capillaries.

## Concluding remarks

**Fig. 5:**
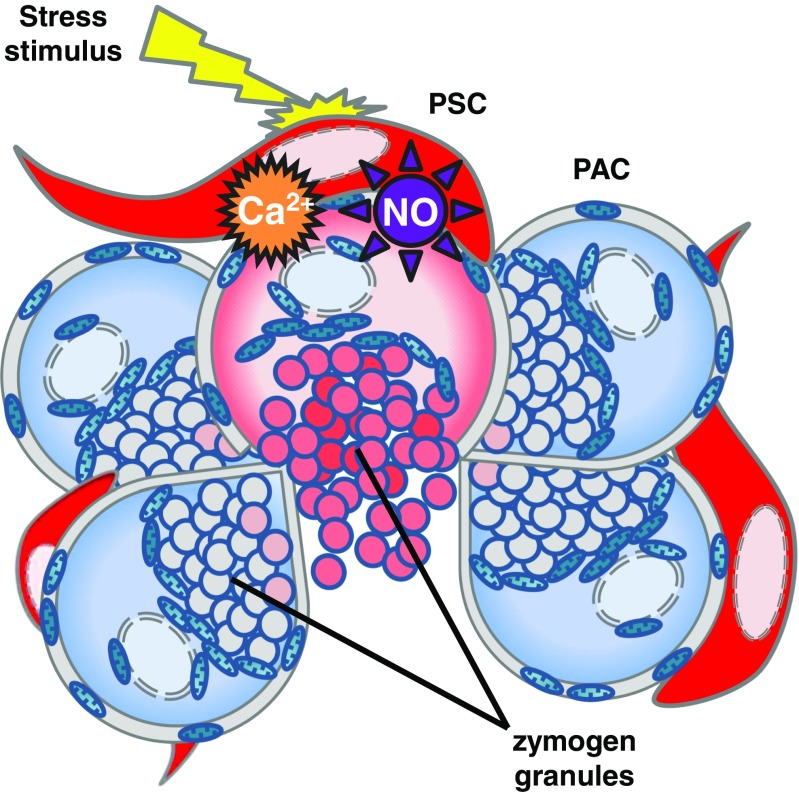
Schematic illustration of a pancreatic lobule. Pathophysiological stimuli (e.g. bile acids, bradykinin, H_2_O_2_) induce stress responses in pancreatic stellate cells (PSC, red), manifested as an increase in the cytosolic Ca^2+^ concentration and NO generation. Stress in PSCs further escalates pathophysiological responses in adjacent pancreatic acinar cells (PAC, blue) leading to premature activation of zymogen granules, exacerbated inflammation and necrotic cell death (*pink*), associated with loss of the cellular content

Initially limited to cancer research, the field of PSCs has extended and now covers diverse aspects of cell biology. Increasingly more attention is directed towards understanding the roles of ion channels, small molecule messengers, such as Ca^2+^ and NO (Fig. 5) as well as retinoids in the physiology of PSCs. Nevertheless, still much has to be learned, especially in relation to the processes that trigger PSC phenotype transition. Given that Ca^2+^ plays a role in activation of other cell types such as lymphocytes [31] or mast cells [23], it would not be at all surprising if Ca^2+^ signals also control the process of phenotype transition in PSCs. Therefore, one of the most exciting challenges in the coming years is detailed understanding of the mechanisms that govern the phenomenon of PSC activation.

Finally, it pays to remember that dysregulated physiology underlies most diseases. Therefore, intensified studies on PSC physiology and the role of Ca^2+^ signalling in these cells may aid the development of novel therapeutic strategies against pancreatic disorders. Particularly important would be proposing new means and approaches to inhibit PSC phenotype transition and thus supress excessive collagen deposition that leads to fibrosis. What is more, development of effective strategies to reverse PSC activation in vivo or to target specifically the population of myofibroblast-like PSCs could be of significant translational perspective.
